# Blood-Based Biomarkers for Predicting the Risk for Five-Year Incident Coronary Heart Disease in the Framingham Heart Study via Machine Learning

**DOI:** 10.3390/genes9120641

**Published:** 2018-12-18

**Authors:** Meeshanthini V. Dogan, Steven R. H. Beach, Ronald L. Simons, Amaury Lendasse, Brandan Penaluna, Robert A. Philibert

**Affiliations:** 1 Department of Biomedical Engineering, University of Iowa, Iowa City, IA 52242, USA; robert-philibert@uiowa.edu; 2 Cardio Diagnostics LLC, 2500 Crosspark Road, Coralville, IA 52241, USA; 3 Department of Psychiatry, University of Iowa, Iowa City, IA 52242, USA; 4 Department of Psychology, University of Georgia, Athens, GA 30602, USA; srhbeach@uga.edu; 5 Department of Sociology, University of Georgia, Athens, GA 30606, USA; rsimons@uga.edu; 6 Information and Logistics Technology Department, University of Houston, Houston, TX 77004, USA; alendass@central.uh.edu; 7 Department of Business Management and Analytics, Arcada University of Applied Sciences, 00560 Helsinki, Finland; 8 Behavioral Diagnostics LLC, 2500 Crosspark Road, Coralville, IA 52241, USA; brandan-penaluna@uiowa.edu

**Keywords:** coronary heart disease, risk prediction, machine learning, epigenetics, genetics, biomarkers, risk factors

## Abstract

An improved approach for predicting the risk for incident coronary heart disease (CHD) could lead to substantial improvements in cardiovascular health. Previously, we have shown that genetic and epigenetic loci could predict CHD status more sensitively than conventional risk factors. Herein, we examine whether similar machine learning approaches could be used to develop a similar panel for predicting incident CHD. Training and test sets consisted of 1180 and 524 individuals, respectively. Data mining techniques were employed to mine for predictive biosignatures in the training set. An ensemble of Random Forest models consisting of four genetic and four epigenetic loci was trained on the training set and subsequently evaluated on the test set. The test sensitivity and specificity were 0.70 and 0.74, respectively. In contrast, the Framingham risk score and atherosclerotic cardiovascular disease (ASCVD) risk estimator performed with test sensitivities of 0.20 and 0.38, respectively. Notably, the integrated genetic-epigenetic model predicted risk better for both genders and very well in the three-year risk prediction window. We describe a novel DNA-based precision medicine tool capable of capturing the complex genetic and environmental relationships that contribute to the risk of CHD, and being mapped to actionable risk factors that may be leveraged to guide risk modification efforts.

## 1. Introduction

Heart disease is responsible for one in every four deaths in the United States [1]. Coronary heart disease (CHD), which is the most common type of heart disease, results in over 350,000 deaths annually. Fortunately, CHD is largely preventable and its associated morbidity and mortality can be reduced if those at high risk can be identified well in advance of an adverse coronary event. Currently, several methods are employed to screen for the risk of incident CHD. The most common approach involves the aggregation of multiple conventional risk factors that are either measured (e.g., cholesterol, diabetes) or self-reported by individuals to their clinicians (e.g., smoking status). Even though there are several multivariate risk calculators, such as the Framingham risk score (FRS) [2] and atherosclerotic cardiovascular disease (ASCVD) risk estimator [3], that combine these factors to assist clinicians in informing the next steps, several studies have reported limitations in the performance and generalizability of these methods [4,5]. Although the reasons for these limitations are multifactorial, in part, the use of unreliable self-report data such as smoking status [6] could affect the performance of these approaches and the downstream decision making process. 

Often, as a part of cardiac risk assessment, the initial clinical consultation is followed by more costly and invasive procedures, such as stress testing and even cardiac angiography [7,8,9]. There are several concerning aspects of these follow-up procedures. First, in particular for cardiac angiography, are the possible complications associated with the invasive nature of the test [10]. Second, even when completed without complications, studies have reported the lack in sensitivity of angiography in identifying at risk individuals with multiple risk factors [11]. Finally, by the time several if not all of these and other tests are completed, an individual would have spent days, if not weeks, undergoing tests prior to obtaining insight into their risk and any guidance on risk modification interventions. 

As a result of these and other challenges, the risk assessment and prevention paradigm for many diseases including heart disease has shifted from more traditional approaches to next-generation “omics” and artificial intelligence based big data approaches that may potentially reduce the cost and time necessary to generate a risk profile. It has become increasingly difficult to ignore the promise these approaches hold in revolutionizing clinical practice, especially with respect to early detection and prevention [12]. To that end, building on the premise that diseases such as CHD stem from the complex interplay between genetic and environmental risk factors, we recently developed an integrated genetic-epigenetic machine learning based framework for assessing the presence of CHD [13]. The incorporation of both genetic and epigenetic biomarkers allow complimentary risk information to be captured simultaneously, especially the confounding effects through gene–environment interactions. In this communication, we adapt and extend this framework to predict the incidence of CHD within five years.

## 2. Materials and Methods

A detailed description of the Framingham Heart Study (FHS) (dbGAP study accession: phs000007) has been provided elsewhere [14,15]. In this study, we used the demographics, and clinical, genetic, and epigenetic data, from the Offspring cohort. Specifically, it included participants who provided a blood sample during the eighth examination cycle, which was conducted between 2005 and 2008, and provided consent to FHS clinical staff to participate in genetics research. The cardiovascular health related outcomes of these individuals were assessed in subsequent examination cycles, resulting in the collection of information on incident CHD status. Data from the Offspring cohort was obtained in a fully anonymized form through dbGAP (https://dbgap.ncbi.nlm.nih.gov). The University of Iowa Institutional Review Board (IRB) approved all analyses described in this study (IRB approval number: 201503802).

Genome-wide DNA methylation (DNAm) data from the Illumina Infinium HumanMethylation450 BeadChip (San Diego, CA, USA) [16] was available for 2567 individuals. Sample and probe level quality control was performed as detailed elsewhere [17], reducing the number of samples to 2560. Of those who passed DNAm quality control, 2406 also had genome-wide genotype data profiled using the Affymetrix GeneChip HumanMapping 500K (Santa Clara, CA, USA) array. Standard quality control on samples and probes was performed in PLINK, as previously described in detail [17]. As a result, data from an additional 111 samples were removed from the dataset. In total, data from 2295 individuals passed both DNAm and genotype quality control. In order to ensure related individuals (i.e., similar genetic makeup) do not influence the training of the prediction model, identity by descent (IBD) of >0.1875 (halfway in between second and third-degree relatives) was used to subset out 696 individuals due to relatedness. This created a natural split for the training and test sets, where those not removed based on IBD (*n* = 1599) made up the training set, while those removed based on IBD (*n* = 696) made up the test set. All DNAm beta values were converted to M-values and scaled to have a zero mean and unit variance. 

For all 2295 individuals whose data passed quality control steps, their conventional risk factors for CHD (age, gender, systolic blood pressure (SBP), high-density lipoprotein (HDL) cholesterol level, total cholesterol level, diabetes status, and self-reported smoking status) at the eighth examination cycle (biomaterial was collected during this cycle) were extracted. Additionally, to determine incident CHD status within five years, the CHD status date was also extracted. Incident CHD was considered present if an individual developed CHD within five years of the eighth examination cycle, while incident CHD was considered absent if an individual was not diagnosed with CHD within five years of the eighth examination cycle. The CHD designation was determined upon review by a panel of three investigators on the Framingham Endpoint Review Committee. Based on this, the final training and test sets included 1180 (19/695 females and 23/485 males diagnosed with clinical CHD within five years) and 524 individuals (8/293 females and 12/231 males diagnosed with clinical CHD within five years), respectively. The conventional risk factors of these individuals are summarized in Table 1. 

As expected, in the training set of 1180 individuals, the number of individuals who were not clinically diagnosed with CHD (1138 individuals) greatly exceeded the number of those who developed CHD within five years (42 individuals). That is approximately a ratio of 1:27. To address this class imbalance and ensure biomarkers are predictive of both the majority and minority classes, we randomly undersampled the majority class to create 27 data subsets. Each data subset consisted of the 42 individuals who were clinically diagnosed with CHD within five years (minority class), and 42 individuals chosen randomly without replacement from the pool of 1138 individuals who were not clinically diagnosed with CHD within five years (majority class). Random undersampling is a common method for handling imbalanced datasets [18]. This made the ratio between the majority and minority classes across all 27 data subsets 1:1.

All variable reduction, variable selection, and modeling steps were only performed on the training set in the form of the 27 data subsets described above. A total of 876,014 loci (403,192 and 472,822 genetic and epigenetic loci, respectively) survived all quality control steps. Our goal in this step of the analysis pipeline was to mine integrated genetic-epigenetic biosignatures that together, are capable of identifying those at high risk for developing symptomatic CHD within five years. To achieve this, we implemented a proprietary integrated genetic-epigenetic biomarker mining algorithm in Python that applies data mining techniques [19] to uncover highly predictive biosignatures from the large number of genetic and epigenetic variables, including non-linear genetic-epigenetic interactions. This included implementing parallelized random sampling of the large number of variables and taking advantage of the capabilities of the Random Forest (RF) algorithm [20], including non-linear interactions between the genetic and epigenetic loci, and bootstrapping to uncover highly predictive biosignatures. Only biosignatures that performed well with respect to metrics such as sensitivity and specificity across all 27 data subsets were retained. As a result, we identified a total of 17 candidate loci (11 single nucleotide polymorphisms (SNPs) and 6 DNAm) that performed with high sensitivity and specificity across all 27 data subsets. 

To determine if we could further reduce and optimize this set of candidate loci while still maintaining the performance across all 27 data subsets, we once again implemented the RF algorithm with different combinations of the 17 candidate loci based on the non-linear interactions between DNAm and SNP pairs. Each combination consisted of at least two SNPs and two DNAm loci, and was evaluated based on the RF sensitivity and specificity across all 27 data subsets. This resulted in a final set of eight loci (4 SNPs and 4 DNAm) that performed well across all 27 data subsets. Once this final variable set was determined, a final RF model consisting of the identified eight loci was fitted for each training data subset, resulting in 27 RF models for five-year CHD risk prediction. Then, the prediction probability cutoff value was tuned in each data subset to maximize both sensitivity and specificity of risk prediction. The final tuned model consisted of 500 trees (ntree) and two variables were randomly sampled as candidates at each split (mtry). 

At the end of the training step, we had 27 trained RF models, all of which consisted of the same eight loci and their respective tuned probability cutoff value for maximizing sensitivity and specificity. All 27 of these models were saved for testing on the test dataset. To determine the classification of each individual in the test dataset, majority voting of the ensemble of 27 models was implemented. Therefore, if at least 14 of the 27 models voted in favor of high risk of symptomatic CHD within five years, then the individual was classified as positive for symptomatic CHD within five years, and vice versa. This binary classification in the test dataset was evaluated using sensitivity, specificity, and F1 score.

To compare the performance of our integrated genetic-epigenetic model to existing CHD risk prediction models, we evaluated the predictive performance of the Framingham risk score [2] and ASCVD risk estimator [3] to identify individuals at high risk for incident CHD (≥20%) on the test dataset. To implement these models, data from the eighth examination cycle was also used. Variables considered by these models include age, gender, total cholesterol, HDL, SBP, diastolic blood pressure (DBP), diabetes status, smoking status, and whether individuals are undergoing blood pressure treatment.

To better understand the association between the genetic and DNAm loci in the integrated genetic-epigenetic model and the conventional CHD risk factors, we regressed these loci against each of the risk factors. This analysis was performed on the training and test sets, combined. A generic version of the regressed equation is provided in Equation (1), where the main and interaction effects are considered. In this equation, the risk factors that were considered include age, gender, total cholesterol, HDL cholesterol, SBP, DBP, hemoglobin A1c, and smoking status. Each of the risk factors (i^th^) was regressed against the main effects of the genetic and epigenetic loci and the interactions between them, where j and k represent the number of genetic and epigenetic loci, respectively. For example, if the risk factor being considered is total cholesterol, and the integrated genetic-epigenetic model contains four SNPs and four DNAm loci, the specific form of this equation is shown in Equation (2). 

Risk Factor_i_~SNP_1_ + … + SNP_j_ + DNAm_1_ + … + DNAm_k_ + SNP_1_ * DNAm_1_ + SNP_j_ * DNAm_k_ (1)

Total Cholesterol~SNP_1_ + SNP_2_ + SNP_3_ + SNP_4_ + DNAm_1_ + DNAm_2_ + DNAm_3_ + DNAm_4_ + SNP1 * DNAm_1_ + SNP_1_ * DNAm_2_ + SNP_1_ * DNAm_3_ + SNP_1_ * DNAm_4_ + SNP_2_ * DNAm_1_ + SNP_2_ * DNAm_2_ + SNP_2_ * DNAm_3_ + SNP_2_ * DNAm_4_ + SNP_3_ * DNAm_1_ + SNP_3_ * DNAm_2_ + SNP_3_ * DNAm_3_ + SNP_3_ * DNAm_4_ + SNP_4_ * DNAm_1_ + SNP_4_ * DNAm_2_ + SNP_4_ * DNAm_3_ + SNP_4_ * DNAm_4_ (2)

## 3. Results

We developed a tool that integrates and aggregates the genetic, lifestyle, and environmental risk factors using whole blood DNA-based biosignatures from 1180 individuals from the FHS Offspring cohort to assess the five-year risk of developing symptomatic CHD. The performance of this developed tool was tested on 524 individuals from the FHS Offspring cohort. The demographics and conventional CHD risk factors of these individuals at the eighth examination cycle are summarized in Table 1. Individuals in the Offspring cohort were of European ancestry. Both the training and test sets consisted of more females than males (~59% and ~56% of females in the training and test sets, respectively). However, more males than females were clinically diagnosed with symptomatic CHD within five years of contributing biomaterial during the eighth examination cycle for the offspring cohort. Males and females clinically diagnosed with symptomatic CHD within five years in the training and test sets were on average older than those not clinically diagnosed with symptomatic CHD within five years. However, in the training set, males diagnosed with symptomatic CHD within five years of the eighth examination cycle, were on average older than females, but vice versa in the test set.

HbA1c and systolic blood pressure (SBP) values were similar for both genders in the training and test sets. However, there were differences in the distribution of other conventional risk factors between males and females, and between those who did and did not develop CHD within five years. For instance, in males total cholesterol was higher in the test set compared to the training set by CHD incidence status, but vice versa in females. As for high density lipoprotein (HDL) cholesterol and diastolic blood pressure (DBP), the average values between the training and test sets were only less similar among the females who developed CHD within five years. Finally, the percent of individuals undergoing treatment for blood pressure at the time of biomaterial collection was greater among those who developed CHD within five years for both males and females.

### 3.1. Integrated Genetic-Epigenetic Prediction Model

We developed a model by integrating single nucleotide polymorphism (SNP) and DNAm data from the 1180 individuals in the training set to predict the risk for symptomatic CHD within five years. This final model is an ensemble of 27 Random Forest (RF) models that were trained and tuned on 27 data subsets, as described in the methods section. The variables contained within each of the 27 models were the eight loci, four SNPs, and four DNAm loci, that were identified using a non-linear data mining algorithm based on their sensitivity, specificity, and area under the receiver operating characteristic curve (AUC) metrics across all 27 data subsets. 

The four DNAm loci included in the model are cg00524912, cg08224787, cg24221633, and cg26119740. The four SNPs retained for prediction are rs2599737, rs6797484, rs7250088, and rs898550. The RF model training performance across all 27 data subsets is summarized in Table 2. The average out-of-bag (OOB) error rate, AUC, sensitivity, and specificity across the 27 models are 0.18 ± 0.04, 0.82 ± 0.04, 0.75 ± 0.06, and 0.73 ± 0.06, respectively.

To determine the performance of the prediction ensemble model on the test dataset, all 27 models were applied on the test dataset of 524 individuals. An individual was considered to be at high risk for developing symptomatic CHD within five years if a majority of the 27 models (i.e., at least 14 of the 27 models) were to vote in favor of it. Of the 524 individuals (20 developed and 504 did not develop symptomatic CHD within five years) in the test dataset, the ensemble model accurately identified the five year risk of 386 (~74%) of the 524 individuals. Among the 20 individuals who were at risk, 14 were identified correctly. Similarly, among the 504 individuals who were not at risk within five years, 372 were identified correctly by the ensemble model. Based on that, the test sensitivity, specificity, and F1 score were 0.70, 0.74, and 0.18, respectively. The performance of the ensemble model in the test dataset was very comparable to its performance in the training set. The confusion matrix for the test set is provided in Table 3. 

**Figure 1:**
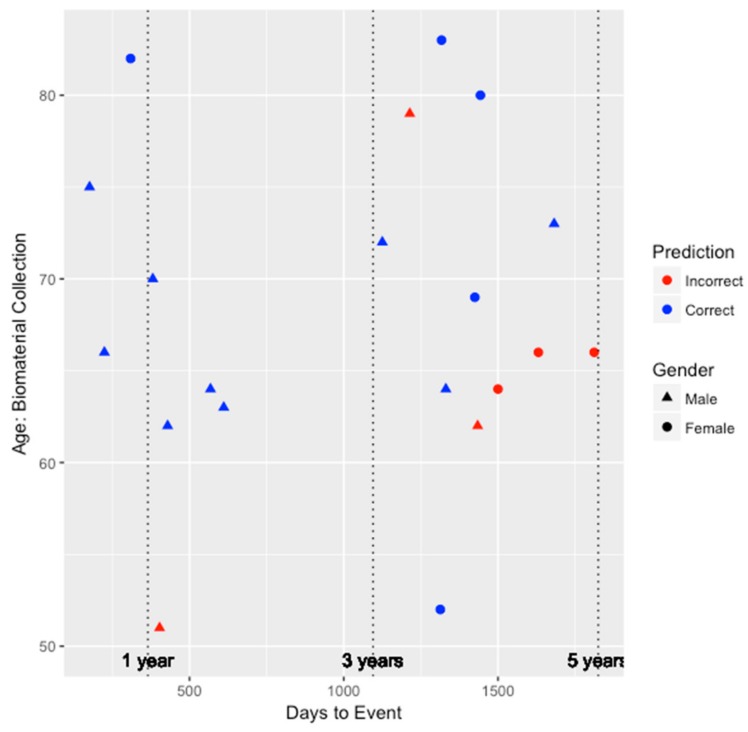
The performance of the integrated genetic-epigenetic tool when identifying those at high risk of symptomatic CHD within five years by age, gender, and days to event.

To better understand the ability of the integrated genetic-epigenetic tool to identify those who were at high risk of symptomatic CHD within five years, we visualized the predictions in the test set with respect to age, gender, and days to symptomatic CHD event, as shown in Figure 1. The average time to event among the 20 individuals in the test set who developed symptomatic CHD within five years is 1016 ± 541 days or 2.78 ± 1.48 years. As seen in Figure 1, the accuracy of the predictions does not appear to be biased by age or gender. Additionally, the tool appears to perform very well (seven out of eight individuals) at identifying those who are at high risk within three years of biomaterial collection. 

### 3.2. Association of Loci to Conventional Coronary Heart Disease Risk Factors

We performed regression analysis of each conventional coronary heart disease (CHD) risk factor on the eight genetic and DNAm loci in the integrated genetic-epigenetic model to better understand the risk factors being captured by these loci. The nominal *p*-value associated with each main and interaction term to each risk factor is summarized in Table 4. Nominal values that were significant at the 0.05 level are shown in bold. Of the total 16 significant associations, only half of those were main effects. Among the eight biosignatures in our final model, no significant main effects were identified for total cholesterol, HDL cholesterol, and HbA1c, whereas no interaction effects were identified for SBP and gender. On the flip side, the main effects of cg26119740 (*PPME1*) and cg24221633 (*KCNQ2*) were significantly associated with more than one risk factor. The only interaction term significantly associated with more than one risk factor is the interaction between rs2599737 (*NUP98*) and cg24221633 (*KCNQ2*). The most significant main effect (nominal *p*-value = 0.003) was between cg26119740 (*PPME1*) and DBP, whereas the most significant interaction (nominal *p*-value = 0.002) was between the rs2599737 (*NUP98*) and cg24221633 (*KCNQ2*) interaction, and DBP as well. 

### 3.3. Comparison with Existing Prediction Models

We compared the performance of our five year risk prediction tool to that of two commonly implemented multivariate approaches, the Framingham risk score (FRS) and the ASCVD risk estimator. Both risk models aggregate several conventional CHD risk factors to infer the risk for developing CHD. Most conventional factors overlap in these two models, with the exception of diastolic blood pressure in the FRS model and the use of blood pressure treatment in the ASCVD Risk Estimator model. The confusion matrices for the FRS and ASCVD risk estimator when implemented on the test dataset are shown in Table 5 and Table 6, respectively. The total number of individuals in these tables is less than the number of individuals in the test set due to missing values and restrictions, such as age needing to be between 40 and 79 years for the ASCVD risk estimator. 

The sensitivity, specificity, and F1 score of prediction in the test set by the FRS model were 0.20, 0.95, and 0.16, respectively. For the ASCVD risk estimator, the test set prediction sensitivity, specificity, and F1 score were 0.38, 0.85, and 0.13, respectively. Clearly, the specificity of these two models outperforms that of the integrated genetic-epigenetic model. In the case of the FRS model, this is because the model is pretty much suggesting that a majority of individuals are not at high risk of developing symptomatic CHD within five years. However, both models drastically underperformed (by 50% for the FRS and 32% for the ASCVD risk estimator) with respect to the more important performance metric, sensitivity, which measures the ability of the model to identify those who actually developed symptomatic CHD within five years. 

**Figure 2:**
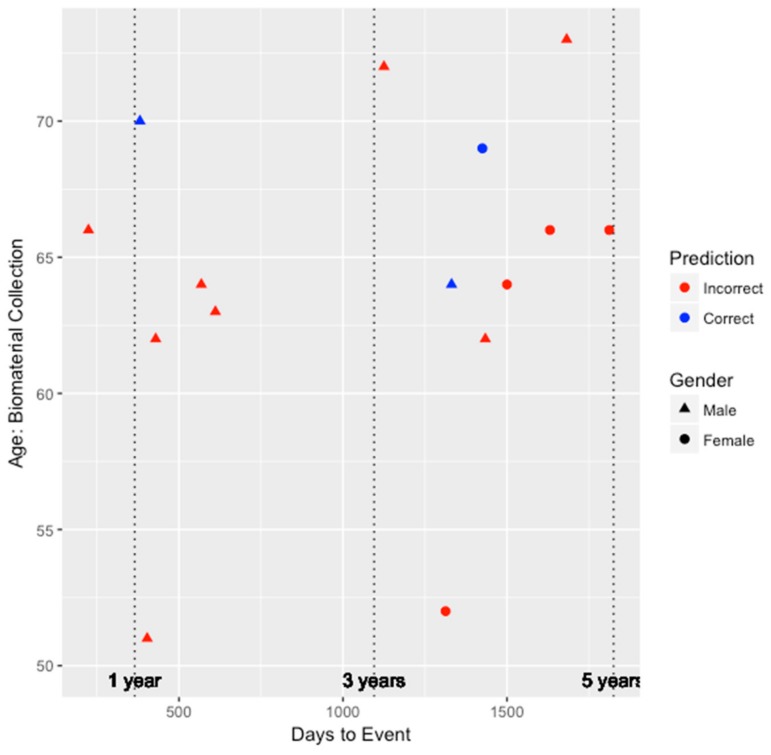
The performance of the Framingham risk score model when identifying those at high risk of symptomatic CHD within five years by age, gender, and days to event.

**Figure 3:**
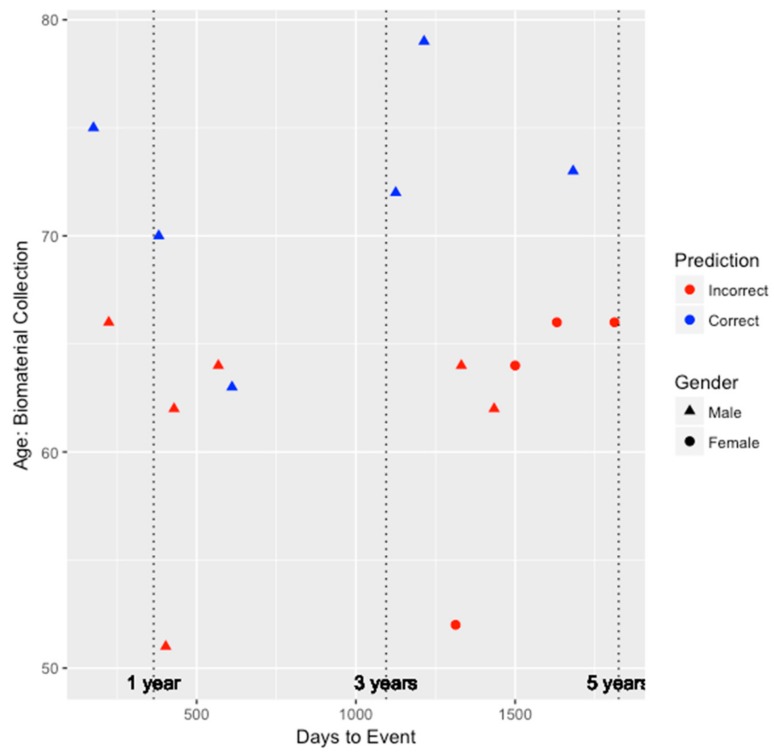
The performance of the ASCVD risk estimator when identifying those at high risk of symptomatic CHD within five years by age, gender, and days to event.

Similar to our genetic-epigenetic tool, we visualized the prediction capability of these two existing models on the validation set by age, gender, and days to event. Figure 2 and Figure 3 depict the performance for the FRS and ASCVD risk estimator models, respectively. Unfortunately, as shown in Figure 2, since the FRS model was only able to predict the risk of three individuals (two males and one female) correctly, we are unable to draw much insight on whether the performance of the model is biased by gender. All three individuals predicted correctly by the FRS model were between the ages of 60 and 70, and two of those three individuals developed CHD within the 3–5 year window since biomaterial collection. However, for the ASCVD risk estimator, as shown in Figure 3, this model tended to perform better among older individuals and for males. Risk prediction for all females was inaccurate. Nevertheless, among the six individuals inaccurately predicted by the integrated genetic-epigenetic model, one of them was correctly identified by the ASCVD risk estimator. 

To further compare the performance amongst the three models, we examined the performance in the 0–3 year window and the 3–5 year window. In the 0–3 year window, the integrated genetic-epigenetic, FRS, and ASCVD risk estimator models accurately predicted the high risk status of seven of eight individuals (87.5%), one of six individuals (16.7%), and three of seven individuals (42.9%), respectively. Similarly, in the 3–5 year window, the integrated genetic-epigenetic, FRS, and ASCVD risk estimator models accurately predicted the high risk status of seven of 12 individuals (58.3%), two of nine individuals (22.2%), and three of nine individuals (33.3%), respectively. The integrated genetic-epigenetic tool and the ASCVD risk estimator performed better in the 0–3 year prediction window, whereas the FRS model did better in the 3–5 year window. However, the integrated genetic-epigenetic model outperformed both conventional risk factor models in both prediction windows. Additionally, the ASCVD risk estimator performed better than the FRS model in both prediction windows. 

## 4. Discussion

The results from our study indicate that a DNA-based tool that combines genetic and epigenetic (environmental and lifestyle) risks for CHD can more sensitively assess the risk of developing symptomatic CHD within five years than currently available conventional risk factor based multivariate risk models. This suggests that, instead of performing and aggregating multiple tests (e.g., lipid panel, HbA1c lab test, blood pressure assessments), our single integrated genetic-epigenetic tool, which requires DNA from only a small amount of blood, can be used directly to predict risk for CHD. Another drawback of using conventional risk factors data is the lack of consistency due to observed variation in some of these assessments throughout the day [21]. 

As previous studies have reported, there are limitations associated with the FRS and ASCVD risk estimator [22,23]. Some limitations of these methods in our study were the low sensitivity observed using the FRS and the lack in performance by the ASCVD risk estimator in females. In contrast, the integrated genetic-epigenetic tool was better at capturing risk in both males and females. However, the use of the FHS Offspring cohort of European ancestry limits our ability to compare and contrast the performance of our tool to that of existing approaches in different ethnic groups. Nevertheless, we are currently working to ensure that our tool is generalizable to all members of our society, and expect to capture ethnic specific genetic and environmental risks for CHD.

While the ability to identify those at risk for CHD well in advance of its manifestation is crucial for prevention, it is just as important for clinicians, once risk is recognized, to guide necessary treatments and interventions in a personalized manner to mitigate risk. As our results demonstrate, encouragingly the integrated genetic-epigenetic biosignatures in the final model map to known genes associated with CHD, and capture variance associated with modifiable risk factors, such as cholesterol and diabetes. Three of the four SNPs and three of the four DNAm sites in the final model map to genes; rs2599737 to Nucleoporin 98 (*NUP98*), rs6797484 to PDZ Domain Containing Ring Finger 3 (*PDZRN3*), rs898550 to Long Intergenic Non-Protein Coding RNA 841 (*LINC00841*), cg26119740 to Protein Phosphatase Methylesterase 1 (*PPME1*), cg00524912 to Intraflagellar Transport 27 (*IFT27*), and cg24221633 to Potassium Voltage-Gated Channel Subfamily Q Member 2 (*KCNQ2*). The other SNP in the final model, rs7250088, is located on chromosome 19, between the Phosphatidylinositol-4-Phosphate 5-Kinase Type 1 Gamma (*PIP5K1C*) gene approximately 5000 base pairs upstream of this SNP, and the Tight Junction Protein 3 (*TJP3*) gene approximately 3000 base pairs downstream of this SNP. Finally, the fourth DNAm site, cg08224787, is located on a chromosome 11 CpG island, between the Olfactory Receptor Family 4 Subfamily C Member 12 (*OR4C12*) and Septin 7 Pseudogene (*LOC441601*) genes.

The roles of some of these biosignatures or their genes in cardiovascular disease in general, or CHD specifically, have been reported in prior studies. For instance, several studies have demonstrated the association of variants in the *LINC00841* gene to myocardial infarction and CHD [24,25]. Shendre and colleagues report that the *LINC00841* gene region, which is associated with myocardial infarction in those of European ancestry, was also significant at the 0.05 level in African Americans [25]. This may suggest that the *LINC00841* gene plays a role in conferring risk for CHD associated myocardial infarction across ethnic groups. Another SNP in our model, rs7250088, was identified as one of the top 150 SNPs associated with CHD using a Random Forest model [26]. A DNAm biosignature gene in our model, *PPME1*, is known to affect the activity of the Protein Phosphatase 2A (*PP2A*) gene, which is a gene that codes for a key phosphatase with multiple cardiac regulatory roles [27,28]. 

Though some of the loci included in the final model may not necessarily be directly associated with CHD, our results suggest that they could be linked to factors that contribute to risk for CHD. The understanding of this mapping of biosignatures to conventional risk factors allows for better targeting of modifiable risk factors that can be leveraged to inform personalized prevention treatments and interventions. This is especially attractive with respect to DNAm signatures, as their dynamic nature can aid in advancing our understanding of the relative changes given an intervention, and in turn to better tailor these interventions and then subsequently monitor change in risk over time. Based on our mapping results (Table 4), we show that both main and interaction effects are significantly associated at the 0.05 nominal level with commonly interrogated risk factors, all of which, besides age and gender, are potentially modifiable. 

It is not surprising that some of the strongest associations for many of these factors were interactions between a SNP and DNAm site. Indeed, it is widely accepted that risk factors for CHD, such as cholesterol, diabetes, and CHD itself, are functions of our genetic variations, environment, and the complex interplay [29,30,31]. For example, one of the stronger associations was observed for the interaction of cg24221633 (*KCNQ*2 gene) with rs2599737 (*NUP98* gene) in the prediction of DBP. Prior studies have shown that potassium channels not only have a central role in cardiac and brain excitability, but could also play a role in the development of CHD risk factors, such as hypertension and diabetes, as well as cardiovascular disease itself [32,33]. The *KCNQ2* gene encodes for one of the five alpha subunits of the voltage-dependent potassium channels (Kv), specifically the Kv7.2 subunit [32]. The *NUP98* gene in the form of the NUP98-HOXA9 fusion protein has been shown to strongly upregulate renin, which is a well-known regulator of blood pressure [34]. Our results suggest that, in addition to a strong association between the main effect of the *KCNQ2* gene to DBP, the *KCNQ2* gene interacting with the *NUP98* gene is also highly associated with DBP. Insights such as these are made possible by non-linear techniques, such as the ones employed in our study for data mining and modeling, which are essential for uncovering highly predictive and novel signatures. 

We are aware that our study has some limitations. While our overall sample size is not too small, the number of incident cases is small, and the individuals in the cohort are only of European ancestry. To address this shortcoming, additional validation studies that have more incident cases and that include participants of diverse ethnic backgrounds are needed. While our integrated genetic-epigenetic tool performed with superior sensitivity (50% and 32% more sensitive than the FRS and ASCVD risk estimator, respectively) to identify those at high risk for incident CHD, it did lack specificity compared to the two multivariate models (21% and 11% less specific than the FRS and ASCVD risk estimator, respectively). Additionally, the integrated genetic-epigenetic model was more sensitive at predicting high risk for CHD in both the 0–3 and 3–5-year windows compared to the other methods. Though it is important for risk assessment tools to perform with high sensitivity and specificity, we believe that given the adverse effect of a false negative, the more important metric is sensitivity. Nevertheless, our goal is to further optimize our model in larger, more diverse cohorts, to improve the specificity and even the sensitivity. 

However, there are several advantages of our integrated genetic-epigenetic tool for assessing the risk of CHD. Firstly, it is a single blood-based test, and therefore does not require the aggregation of several assessments. Our study indicates that this tool is more sensitive than conventional risk-factor based approaches, and performs well in both males and females. The biosignatures are capable of capturing the complex genetic and lifestyle relationships that contribute to the risk for CHD. In addition to elucidating novel gene–environment relationships that can be leveraged to assess the risk for CHD, the mapping of the environmental component to modifiable risk factors could aid in informing personalized prevention interventions and monitoring changes in risk over time.

## 5. Patents

On behalf of Drs. Dogan and Philibert, the University of Iowa has filed intellectual property claims with respect to the content of this article (US Patent application 62,455,416: Compositions and Methods for Detecting Predisposition to Cardiovascular Disease).

## Figures and Tables

**Table 1:** Summary of demographics and conventional coronary heart disease (CHD) risk factors at the eighth examination cycle for the 1180 and 524 individuals in the training and test sets, respectively.

	Training (*n* = 1180)		Test (*n* = 524)	
	CHD ^1^	No CHD ^2^	CHD ^1^	No CHD ^2^
**Gender (count)**				
Male	23	462	12	219
Female	19	676	8	285
**Age (years)**				
Male	70.8 ± 9.7	65.2 ± 8.1	66.8 ± 7.5	61.6 ± 8.6
Female	68.5 ± 9.0	65.6 ± 8.2	70.3 ± 10.7	63.8 ± 9.1
**Total Cholesterol (mg/dL)**				
Male	166 ± 50	178 ± 32	173 ± 34	185 ± 32
Female	204 ± 50	201 ± 36	181 ± 38	196 ± 33
**HDL Cholesterol (mg/dL)**				
Male	48 ± 15	50 ± 14	49 ± 17	51 ± 15
Female	53 ± 16	66 ± 19	60 ± 19	66 ± 19
**HbA1c (%)**				
Male	5.8 ± 0.4	5.7 ± 0.8	5.7 ± 0.7	5.6 ± 0.5
Female	5.9 ± 0.8	5.7 ± 0.5	5.6 ± 0.4	5.6 ± 0.5
**SBP (mmHg)**				
Male	134 ± 17	130 ± 17	138 ± 25	128 ± 16
Female	137 ± 19	128 ± 18	135 ± 24	125 ± 17
**DBP (mmHg)**				
Male	73 ± 11	77 ± 10	75 ± 8	78 ± 9
Female	77 ± 9	73 ± 10	66 ± 10	73 ± 10
**Smoker (count)**				
Male	1 (4%)	29 (6%)	3 (25%)	12 (5%)
Female	2 (11%)	49 (7%)	0 (0%)	28 (10%)
**Blood Pressure Treatment (count)**				
Male	16 (70%)	223 (48%)	7 (58%)	86 (39%)
Female	10 (53%)	250 (37%)	4 (50%)	114 (40%)

^1^ Those who developed symptomatic coronary heart disease (CHD) within five years of contributing biomaterial during the Offspring cohort eighth examination cycle. ^2^ Those who did not develop symptomatic CHD within five years of contributing biomaterial during the Offspring cohort eighth examination cycle. HDL: high-density lipoprotein, SBP: systolic blood pressure, DBP: diastolic blood pressure.

**Table 2:** Integrated genetic-epigenetic Random Forest model prediction performance in the training set.

Model	OOB Error Rate	AUC	Sensitivity	Specificity
1	0.24	0.76	0.71	0.67
2	0.22	0.78	0.67	0.68
3	0.19	0.81	0.71	0.71
4	0.20	0.80	0.76	0.74
5	0.13	0.87	0.88	0.74
6	0.22	0.78	0.69	0.67
7	0.17	0.83	0.74	0.72
8	0.10	0.90	0.81	0.86
9	0.15	0.85	0.76	0.74
10	0.11	0.89	0.88	0.76
11	0.14	0.86	0.81	0.74
12	0.14	0.86	0.74	0.80
13	0.13	0.87	0.79	0.77
14	0.20	0.80	0.74	0.74
15	0.18	0.82	0.71	0.77
16	0.22	0.78	0.71	0.70
17	0.20	0.80	0.71	0.76
18	0.24	0.76	0.67	0.63
19	0.19	0.81	0.74	0.76
20	0.23	0.77	0.74	0.56
21	0.20	0.80	0.76	0.70
22	0.18	0.82	0.79	0.79
23	0.27	0.73	0.67	0.72
24	0.10	0.90	0.86	0.81
25	0.18	0.82	0.81	0.67
26	0.17	0.83	0.79	0.72
27	0.19	0.81	0.69	0.74

OOB: out-of-bag; AUC: area under the receiver operating characteristic curve

**Table 3:** The confusion matrix of the integrated genetic-epigenetic ensemble of 27 models on the test dataset consisting of 524 individuals for predicting the five-year risk of developing symptomatic CHD.

	Predicted	
True	Not at High Risk	At High Risk
**Did not develop symptomatic CHD within 5 years**	372	132
**Developed symptomatic CHD within 5 years**	6	14

**Table 4:** The nominal *p*-value of the main and interaction terms of genetic and epigenetic biosignatures to conventional CHD risk factors.

Locus	Total Cholesterol	HDL Cholesterol	HbA1c	Smoking Status	SBP	DBP	Age	Gender
rs2599737	0.94	0.81	0.55	0.59	0.29	0.95	0.28	0.28
rs6797484	0.27	0.86	0.59	0.15	0.006	0.21	0.06	0.62
cg26119740	0.68	0.74	0.20	0.98	0.47	0.003	0.007	0.02
cg00524912	0.67	0.91	0.91	0.88	0.82	0.33	0.06	0.05
rs898550	0.40	0.17	0.27	0.80	0.05	0.75	0.79	0.25
cg24221633	0.47	0.56	0.07	0.03	0.12	0.007	0.45	0.20
cg08224787	0.18	0.12	0.71	0.61	0.85	0.53	0.66	0.40
rs7250088	0.80	0.67	0.36	0.02	0.64	0.83	0.44	0.18
rs2599737: cg26119740	0.04	0.67	0.55	0.15	0.41	0.20	0.59	0.29
rs2599737: cg00524912	0.25	0.03	0.94	0.67	0.49	0.07	0.88	0.34
rs2599737: cg24221633	0.94	0.32	0.38	0.06	0.65	0.002	0.01	0.09
rs2599737: cg08224787	0.68	0.08	0.82	0.78	0.50	0.53	0.36	0.54
rs6797484: cg26119740	0.41	0.36	0.01	0.57	0.68	0.09	0.12	0.37
rs6797484: cg00524912	0.04	0.54	0.10	0.16	0.97	0.96	0.29	0.97
rs6797484: cg24221633	0.41	0.50	0.84	0.05	0.11	0.56	0.21	0.97
rs6797484: cg08224787	0.63	0.49	0.17	0.19	0.95	0.74	0.35	0.27
rs898550: cg26119740	0.06	0.99	0.003	0.59	0.72	0.85	0.36	0.97
rs898550: cg00524912	0.66	0.13	0.55	0.88	0.39	0.59	0.32	0.32
rs898550: cg24221633	0.45	0.09	0.09	0.26	0.55	0.49	0.86	0.15
rs898550: cg08224787	0.08	0.14	0.87	0.55	1.00	0.48	0.72	0.26
rs7250088: cg26119740	0.35	0.92	0.71	0.89	0.13	0.26	0.98	0.31
rs7250088: cg00524912	0.81	0.44	0.88	0.98	0.79	0.79	0.61	0.48
rs7250088: cg24221633	0.46	0.05	0.68	0.35	0.22	0.63	0.71	0.20
rs7250088: cg08224787	0.81	0.22	0.84	0.89	0.40	0.95	0.43	0.59

**Table 5:** The confusion matrix of the Framingham risk score on the test dataset for predicting a high risk of developing symptomatic CHD within five years.

	Predicted	
True	Not at High Risk	At High Risk
**Did not develop symptomatic CHD within 5 years**	423	20
**Developed symptomatic CHD within 5 years**	12	3

**Table 6:** The confusion matrix of the ASCVD risk estimator on the test dataset for predicting a high risk of developing symptomatic CHD within five years.

	Predicted	
True	Not at High Risk	At High Risk
**Did not develop symptomatic CHD within 5 years**	382	69
**Developed symptomatic CHD within 5 years**	10	6
